# Human biomonitoring on heavy metals in Ath: methodological aspects

**DOI:** 10.1186/0778-7367-69-10

**Published:** 2011-12-05

**Authors:** Javiera Rebolledo, Sebastien Fierens, Ann Versporten, Ethel Brits, Pierre De Plaen, An Van Nieuwenhuyse

**Affiliations:** 1 Direction of Public Health and Surveillance, Health and Environment Service, Scientific Institute of Public Health (IPH), Brussels, Belgium; 2 Department of Public Health, Section of Occupational, environmental and Insurance Medicine, Katholieke Universiteit Leuven, Leuven, Belgium

**Keywords:** Ath, biomonitoring, heavy metals, methodology, sampling, study design

## Abstract

The municipality of Ath is characterised by the presence, in its center, of two non-ferrous metal industries whose emissions make local residents concerned for their health. Therefore, authorities of the Walloon Region and the municipality of Ath undertook biomonitoring to assess the impact of those industrial emissions on heavy metal body burden in humans.

This paper describes the study design and methodology used to carry out this human biomonitoring.

A random sampling was done in the general population, in two areas of Ath: an area centered around the industries and a peripheral area. The target population was children (2.5-11 years) and adults (40-60 years) without occupational exposure. The three-stage sampling procedure consisted of a mixture of both mail and telephone recruitment. Firstly, 3259 eligible people, identified from a population register, were mailed an introductory letter. In a second stage, eligible individuals were contacted by phone to propose them to participate in the study. They were randomly contacted until the required sample size was obtained. In the third stage, a second mail was sent to those who agreed to participate with a questionnaire to be filled out. Finally, biological samples (blood and urine) from 278 persons were collected. The final participation rate of this study was 24%.

This sampling procedure, especially designed for the purpose of this biomonitoring study in Ath, allowed us to recruit a sample representative of the population of children and adults of Ath, reaching the expected sample size in a short period of time.

## Introduction

The Belgian municipality of Ath is characterised by the presence, in the center of the town, of two industries of non-ferrous metals. The industries are known for their production of lead, cadmium, chromium, cobalt, nickel and zinc. Therefore, Ath may have long been polluted by the emissions of those industries. Furthermore, an environmental study had found relatively high levels of lead, cadmium, chromium, cobalt and nickel in the surface soil in the vicinity of these industries [1]. Health concerns rose among neighbors of this area due to the possible exposure to heavy metals and exposure-related health effects, especially for children living close to such sources. Therefore, the authorities of the Walloon Region and the municipality of Ath decided to undertake a study to assess the impact of those industrial emissions on heavy metal burden in the human body, and their possible health effects.

This study consisted of a human biomonitoring, measuring lead, cadmium, chromium, cobalt and nickel concentrations in biological samples. The results of this biomonitoring are extensively reported in another publication (Fierens et al., *Human biomonitoring of heavy metals in the vicinity of non-ferrous metal industries in Ath, Belgium*, submitted). The present paper addresses the methodology selected to conduct a study that allowed us to investigate the impact on age groups likely to be most susceptible, and to compare heavy metal levels depending on the exposure, taking into account and ensuring the representativeness of the Ath population [2-4].

## Study zone

Two geographical areas, central and peripheral, were defined within the limits of the municipality of Ath, based on the proximity to the industries and the potential exposure to heavy metals.

*The central area*, presumed to be the area where the population was most exposed to heavy metals, was defined by circles of a one kilometer radius around each industry, with the industries located ± 300 meters apart.

*The peripheral area*, where the population is theoretically less or not exposed to heavy metals, consisted of several villages selected in the periphery of the municipality. These villages were chosen based on four criteria: *i*) a distance greater than 3 kilometers from the industries; *ii*) the absence of other specific sources of exposure; *iii*) out of the prevailing wind direction (South-west); and *iv*) large enough number of people for the sampling. The selected villages were Hautaing, Ligne, Mainvault, Moulbaix, Ormeignies, and Ostiches.

An extension of the geographical areas was possible in order to obtain a larger population in case the sample size was not reached in the first step of the sampling.

## Study population

The target populations were children and adults. Among children, two subgroups were defined: 2.5 to 6 years of age and 7 to 11 years of age. In order to perform less invasive investigations in the subgroup of 2.5 to 6 years, which is particularly vulnerable to lead [5-8], only lead was measured in this group. In the age group of 7 to 11 years, we could perform all investigations. That is why we subdivided the youngest age group. Adults studied were aged 40 to 60 years. This choice was motivated by the following facts: a) for cumulative metals, time of exposure and consequently the age of exposed people are determinant factors [9,10]; b) peak concentration of cadmium in kidneys is usually observed in people aged 50 to 60 years; and c) the limit of 60 years was chosen because of higher co-morbidity after this age.

Besides the geographical area and the age, another inclusion criterion was the residence length in the study area, which had to be at least 10 years for adults, 1 year for children aged 7 to 11 years, and 6 months for children aged 2.5 to 6 years.

Since this is an environmental exposure study, occupationally exposed people have been excluded from the study.

## Power calculation

The sample size calculation for continuous variables approach was used. This approach calculates a sample size that will return values, in percentage, significantly higher than a reference value. The average reference value used is the blood lead value obtained from available data in the Flemish population.

After calculation for a difference of 30%, the sample size was as follows: 48 children from 2.5 to 6 years; 38 children from 7 to 11 years; 56 adults. In order to take into account both the central and the peripheral areas defined for the study, the sample size calculated for each group of age was doubled. The total sample expected was then: n = 284 (96 + 76 + 112) people.

## Sampling procedure

### Preparation

The municipal administration of Ath provided an exhaustive population database according to our request based on age categories, length of residence in Ath and village of residence. From this database, extensive data lists of people who met the sampling criteria of age and length of residence in Ath, were developed for the two geographical areas. These data lists were developed following the three age group classification and stratified by sex for adults. Due to the use of phonecall recruitment, only those people with an available landline telephone number were selected. Finally, a total of 3259 eligible people remained in the lists. For each of the lists, the order of potential participants was drawn at random. Individuals could then be contacted in the order of the list until the required number of participants was reached.

Through this recruitment procedure we assumed that people with a landline telephone were representative of the whole population of Ath. It is possible that a segment of the population was missed. Therefore a selection bias cannot be excluded.

### Recruitment

**Figure 1:**
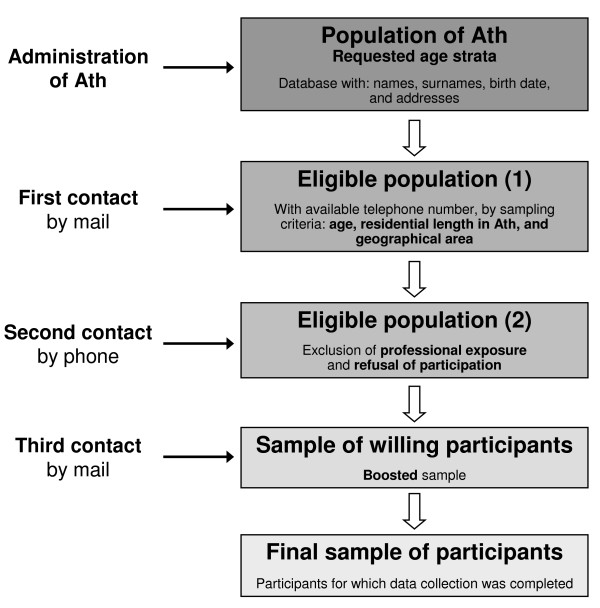
**Sample design and recruitment procedure of the biomonitoring in Ath, 2009**.

The sample recruitment was done in a three-stage procedure (Figure 1). In the first stage, an introductory letter was sent by mail to the 3259 eligible people explaining them the study aims and procedure, so they would not be surprised when contacted by phone to participate in the study as a result of drawing lots.

In the second stage, people previously reached by mail were contacted by phone to ask them to participate in the study. For this, professional interviewers followed a "call procedure" previously defined. Occupationally exposed people were excluded at this stage. An appointment was set up for the data collection session for people who agreed to participate in the study.

Eligible individuals were contacted until the expected sample size was reached. The interviewers made 2606 calls: 1178 were "valid" contacts (when someone of the household answered) and 1428 contacts were "invalid" (unanswered calls, answering machine, faxes or wrong numbers). Of the 1178 people contacted, 832 refused to participate in the study and 33 were excluded based on exclusion criteria. The sample was boosted to 313 people, corresponding to a 10% extra compared to the expected sample size, in order to anticipate any absence from the sample-taking session.

In the third stage, a second mail was sent one week prior to the data collection session, to the 313 people who accepted to participate in the study. This mail confirmed the scheduled appointment and contained a full explanatory document, an informed consent document to be signed, and a questionnaire to be filled out.

Finally, 278 people actually came to the appointment and provided biological samples, of which 98 were children from 2.5 to 6 years, 74 children aged 7 to 11 years and 106 adults (54 women and 52 men). Therefore, the final participation rate of this study was 24% (278/(1178 - 33)).

## Data collection

The data collection sessions were conducted between February 26^th ^and March 20^th ^2009 and took place in a public location in Ath. In order to standardise the data collection, all the sessions were carried out in one and the same place by one single team composed of a nurse, a doctor, and two research collaborators. The data collection session included biological specimen collection and receipt of the filled out questionnaire.

### Biological specimen collection

Blood and urine specimens were collected from all consenting survey participants except for children from 2.5 to 6 years, for whom only a small blood specimen sample was collected from a slight prick in the finger tip. This is a streamlined and less invasive procedure. For the rest of the participants, approximately 10 ml of venous blood were drawn and a sample of minimum 10 ml of urine was collected. After each session, specimens were shipped to the laboratory for analysis. Standard protocols were applied for all procedures.

### The filled out questionnaire

Two different questionnaires, one for adults and one for children, were developed. They consisted of a series of questions designed to obtain necessary information to interpret the blood and urine results, including socio-demographic information, questions relating to surroundings and the environment, lifestyle and behavior of participants, health status, and food consumption.

## Ethical aspect

This study was approved by the Ethics Commission of the Medicine Faculty of the Université catholique de Louvain (UCL). In addition, a statement was submitted to the Belgian Commission for the Protection of Privacy regarding the processing of personal data.

## Notification and communication of the findings

All participants were notified of their examination findings. When wished by the participant, the results were also sent to their general practitioner. Participants with levels above the reference level (95^th ^percentile in general population) were strongly advised to contact their general practitioner. The results of the whole study were presented in a public session and were planned to be reported in scientific journals.

## Conclusions

A three-stage sampling procedure, based on a combination of both mail and telephone recruitment, was carried out for a human biomonitoring study of heavy metals in Ath. The study design defined three age groups (non professionally exposed adults and children) in two geographic areas (center and periphery). The sample recruitment resulted in a final sample of 278 participants and a response rate of 24%. The sampling procedure, especially designed for this biomonitoring in Ath, allowed us to recruit a representative sample of the population of children and adults of Ath, reaching the expected sample size in a short period of time.

## Competing interests

The authors declare that they have no competing interests.

## Authors' contributions

JR participated in the design of the study, in the field work, and wrote the manuscript. SF participated in the design of the study, in the field work, revised the manuscript, and participated in the coordination of the study. AV participated in the conception and design of the study, and in the field work. EB participated in the design of the study, particularly for the development of the questionnaires. PDP participated in the conception and design of the study. AVN participated in the conception, the design, and the coordination of the study. All authors read and approved the final manuscript.
